# Antibiotic resistance surveillance of *Klebsiella pneumoniae* complex is affected by refined MALDI-TOF identification, Swiss data, 2017 to 2022

**DOI:** 10.2807/1560-7917.ES.2022.27.45.2200104

**Published:** 2022-11-10

**Authors:** Irene Katharina Voellmy, Claudia Lang, Michael Gasser, Andreas Kronenberg, on behalf of the Swiss Centre for Antibiotic Resistance (ANRESIS)

**Affiliations:** 1 Swiss Centre for Antibiotic Resistance ANRESIS, Institute for Infectious Diseases, University of Bern, Bern, Switzerland; 2 Viollier AG, Allschwil, Switzerland

**Keywords:** *Klebsiella pneumoniae* complex, *Klebsiella variicola*, ANRESIS, antimicrobial resistance, surveillance

## Abstract

**Background:**

Modern laboratory methods such as next generation sequencing and MALDI-TOF allow identification of novel bacterial species. This can affect surveillance of infections and antimicrobial resistance. From 2017, increasing numbers of medical microbiology laboratories in Switzerland differentiated *Klebsiella variicola* from *Klebsiella pneumoniae* complex using updated MALDI-TOF databases, whereas many laboratories still report them as *K. pneumoniae* or *K. pneumoniae* complex.

**Aim:**

Our study explored whether separate reporting of *K. variicola* and the *Klebsiella pneumoniae* complex affected the ANRESIS surveillance database.

**Methods:**

We analysed antibiotic susceptibility rates and specimen types of *K. variicola* and non-*K. variicola*-*K. pneumoniae* complex isolates reported by Swiss medical laboratories to the ANRESIS database (Swiss Centre for Antibiotic Resistance) from January 2017 to June 2022.

**Results:**

Analysis of Swiss antimicrobial resistance data revealed increased susceptibility rates of *K. variicola* compared with species of the *K. pneumoniae* complex other than *K. variicola* in all six antibiotic classes tested. This can lead to underestimated resistance rates of *K. pneumoniae* complex in laboratories that do not specifically identify *K. variicola*. Furthermore, *K. variicola* strains were significantly more often reported from blood and primarily sterile specimens than isolates of the *K. pneumoniae* complex other than *K. variicola,* indicating increased invasiveness of *K. variicola*.

**Conclusion:**

Our data suggest that refined differentiation of the *K. pneumoniae* complex can improve our understanding of its taxonomy, susceptibility, epidemiology and clinical significance, thus providing more precise information to clinicians and epidemiologists.

**Key public health message**

**What did you want to address in this study?**

Our previously known microorganism species classification is increasingly refined by novel technological methods. In this study, we investigated how this technological leap affects antibiotic resistance surveillance data using *Klebsiella pneumoniae* as an example.

**What have we learnt from this study?**

The introduction of further differentiation of the *Klebsiella pneumoniae* species complex into individual species was inconsistent across laboratories. This affects antibiotic resistance monitoring: resistance rates of the species complex as a whole appear lower in those laboratories where *Klebsiella variicola*, one of the less resistant species in the complex, is still included.

**What are the implications of your findings for public health?**

Harmonisation of resistance monitoring requires early identification of such inhomogeneous data and agreement on a common approach, otherwise resistance data may differ across countries (and even within countries) for methodological reasons.

## Introduction

DNA sequencing methods refine phylogenetic population structures and allow identification of novel species. Novel species may show distinct resistance rates and clinical significance, revising our understanding of epidemiology, ecology, pathogenesis and antimicrobial susceptibility [1,2]. Examples include *Staphylococcus argenteus*, *Gemella taiwanensis*, *Actinotignum* spp [1], *Macrococcus* spp [2], which have been identified as novel sources of infections. The taxonomies of *Corynebacteriae* and *Enterobacterales* have been reclassified considerably, with new species and subspecies [2]. Furthermore, colistin-resistant *Citrobacter europaeus* strains and multi-resistant *Enterobacter bugandensis* isolates with the potential of increased virulence have been described [2]. In addition to molecular tools, matrix-assisted laser desorption ionization-time of flight mass spectrometry (MALDI-TOF MS) can reliably identify a growing number of species in routine diagnostics, which allows treatment adjustment and increased understanding of clinical consequences in practical healthcare [3]. A recent study has shown the potential of MALDI-TOF to identify subgroups within a bacterial species for epidemiological assessments [4].

The *Klebsiella pneumoniae* complex is highly diverse: as a member of *Enterobacterales*, this complex of closely related species occupies various ecological niches. Several virulence and resistance factors have been described in strains causing a variety of infections in humans and animals [5]. To date, phylogenetic research on the population structure of the *K. pneumoniae* complex has identified four species with a total of seven subspecies [6]. Recently, an additional species has been proposed [7]: ‘*K.* *quasivariicola’*. 

*Klebsiella pneumoniae* sensu stricto has been isolated from asymptomatic humans, but is also a frequent cause of serious invasive infections associated with strains carrying large varieties of virulence or resistance accessory genes [5]. Other *Klebsiella* species are considered less virulent and resistant, although recent studies applying refined species discrimination methods revealed virulence and antibiotic resistance genes also in these species [7]. For example, *K. variicola* has been recovered from a wide range of environments and hosts [8]. It is an opportunistic human pathogen causing e.g. bloodstream, respiratory and urinary tract infections [9]. Moreover, studies report genetic potential of virulence traits [10], increased clinical virulence [11] and antibiotic resistance [12].

Increasing antimicrobial resistance rates are a concern for public health [13]. Many countries, including Switzerland, address this issue by antibiotic stewardship guidelines and surveillance databases to control resistance rates. However, data quality and comparability are very important to provide meaningful surveys on a national and international level, while appropriately integrating novel findings such as the identification of novel species, emerging resistance genes or virulence factors. Since 2017, commercial MALDI-TOF databases have enabled differentiation of *K. variicola* from other species of the *K. pneumoniae* complex. An increasing number of laboratories has started reporting *K. variicola* separately and report the remaining species of the *K. pneumoniae* complex as *K. pneumoniae* or *K. pneumoniae complex*. Thus, *K. pneumoniae* or *K. pneumoniae complex* can include different sets of species depending on the reporting policies of individual laboratories. For antimicrobial resistance surveillance databases, inconsistent reporting practices may affect trend analyses of resistance rates if novel species show differential antimicrobial susceptibilities.

The Swiss Centre for Antibiotic Resistance (ANRESIS) has hosted the Swiss national antimicrobial resistance surveillance database since 2004 and collects antibiotic resistance data of all bacterial species isolated and reported from various invasive and non-invasive specimens (primary sterile specimens, swabs, blood cultures, urine, sputum, stool) of up to 37 medical diagnostic laboratories (in 2020). Reported isolates were recovered from patients treated in different facilities, ranging from tertiary hospitals to general practices. The contributing laboratories are widely distributed across Switzerland, representing 89% of all hospitalised patients [14]. With the emergence of reported *K. variicola* isolates, the aim of this study was to investigate whether differentiation of *K. variicola* from other species of the *K. pneumoniae* complex (we refer to those as non-*K. variicola-K. pneumoniae*-complex (non-kv-kpc) in this article) may affect the ANRESIS surveillance database. 

## Methods

We extracted susceptibility rates and specimen types of *K. variicola* and non-kv-kpc isolates from the ANRESIS database for all laboratories differentiating *K. variicola* from *K. pneumoniae* complex, starting from 1 January 2017 when *K. variicola* became available in the MALDI-TOF database for routine diagnostics, or as soon as individual laboratories began *K. variicola* differentiation, until 13 June 2022. Species identification and antimicrobial susceptibility tests (AST) were performed at local laboratories following the guidelines from European Committee on Antimicrobial Susceptibility Testing (EUCAST) (36 laboratories) or the Clinical and Laboratory Standards Institute (CLSI) (one laboratory until 2020). The individual laboratories used various AST methods, such as automated minimum inhibitory concentration (MIC) tests or disc diffusion tests measuring inhibition zones manually or through an automated process. All reporting laboratories are accredited by the Swiss Accreditation Service (SAS). 

Resistance categories were defined resistant if categorised R (resistant) by reporting laboratories, and non-resistant if categorised S (susceptible) or I (susceptible, increased exposure), according to EUCAST's revised susceptibility categorisation that has been introduced gradually in Swiss laboratories since 2019 [15]. For third- and fourth-generation cephalosporins, we considered isolates resistant if at least one substance of this class was categorised R. Our database included the following third- and fourth-generation cephalosporins: cefepime, cefixime, cefotaxime, cefpodoxime, ceftazidime, ceftibuten and ceftriaxone. For carbapenems, we only considered meropenem and imipenem, and rated them resistant if they were categorised as R, because many laboratories did not include ertapenem in their AST panels. For all remaining antibiotic classes, we included the most commonly reported substance in the analysis: amoxicillin-clavulanic acid, ciprofloxacin, gentamicin and trimethoprim-sulfamethoxazole, resulting in a total of six antibiotic classes tested. 

Invasive strains were defined as isolates obtained from blood or primarily sterile specimen types, such as cerebrospinal fluid (CSF) and tissue specimens. We compared susceptibility rates and proportions of invasive strains of *K. variicola* and non-kv-kpc using Fisher's exact test. Repeated testing was accounted for by applying sequential Bonferroni corrections. All statistical tests were conducted in R v4.2.1 [16].

## Results

Laboratories routinely identifying *K. variicola* increased from four of 30 laboratories reporting antibiotic susceptibility data to ANRESIS in 2017 to 18 of 35 laboratories in June 2022. *Klebsiella variicola* was less frequently isolated than non-kv-kpc isolates: from 1 January 2017 to 13 June 2022, 14.1% (n = 9,899) of the 70,114 isolates analysed were reported as *K. variicola*. All six antibiotic substances or antibiotic classes tested showed significantly higher susceptibility rates in *K. variicola* than in non-kv-kpc isolates. These differences remained after applying sequential Bonferroni corrections (Table and Figure) and after considering EUCAST's previous definition of ‘non-susceptibility’ (isolates tested I (intermediate) or R (resistant)). See Supplementary Table S1 for the detailed results of Fisher’s exact tests after applying EUCAST’s previous definition of ‘non-susceptibility’. 

**Table:** Resistance rates against six antibiotics of *Klebsiella variicola* and non-kv-kpc isolates collected by ANRESIS, Switzerland, 1 January 2017–13 June 2022 (n = 70,114)

Antibiotic	Non-kv-kpc		*Klebsiella variicola*		OR	95% CI	p value
	Resistant (%)	n (total)	Resistant (%)	n (total)			
Amoxicillin-clavulanic acid	9.1	60,114	4.7	9,889	2.0	1.8–2.2	< 0.001
Carbapenem	0.4	45,460	0.1	7,832	7.7	3.0–28.5	< 0.001
Cephalosporin 3rd/4th generation	6.6	54,530	2.9	8,883	2.4	2.1–2.7	< 0.001
Ciprofloxacin	6.9	59,743	2.1	9,856	3.4	2.9–3.9	< 0.001
Gentamicin	2.8	37,806	1.7	6,447	1.6	1.3–2.0	< 0.001
Trimethoprim-sulfamethoxazole	9.5	58,489	3.9	9,542	2.6	2.3–2.9	< 0.001

ANRESIS: Swiss Centre for Antibiotic Resistance; CI: confidence interval; non-kv-kpc: non-*Klebsiella variicola-Klebsiella pneumoniae*-complex; OR: odds ratio.

Fisher’s exact tests were used and p values adjusted using sequential Bonferroni corrections. Non-kv-kpc refers to all species of the *K. pneumoniae* complex except *K. variicola*. Carbapenem includes imipenem and meropenem, cephalosporin 3rd/4th generation includes all third- and fourth-generation cephalosporins available in the ANRESIS database (i.e. cefepime, cefixime, cefotaxime, cefpodoxime, ceftazidime, ceftibuten and ceftriaxone). Resistant isolates consist of all isolates categorised resistant (R) by reporting laboratories.

**Figure:**
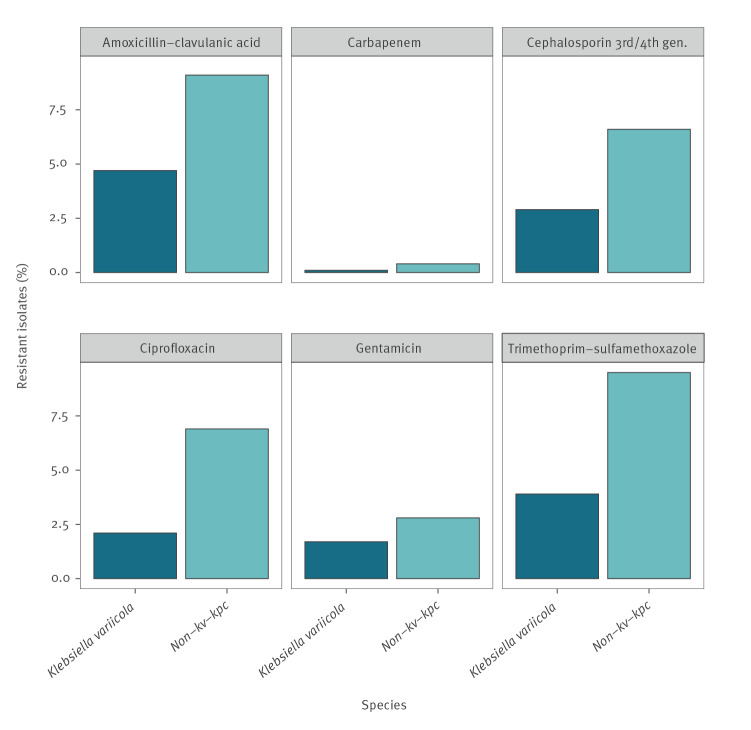
Antibiotic resistance rates in *Klebsiella variicola* isolates (n = 9,899) vs non-kv-kpc isolates (n = 60,215) collected by ANRESIS, Switzerland, 1 January 2017–13 June 2022

ANRESIS: Swiss Centre for Antibiotic Resistance; gen.: generation; non-kv-kpc: non-*Klebsiella variicola*-*Klebsiella pneumoniae*-complex.

Non-kv-kpc refers to all species of the *K. pneumoniae* complex except *K. variicola*. Carbapenem includes imipenem and meropenem, cephalosporin 3^rd^/4^th^ gen. includes all third- and fourth-generation cephalosporins available in the ANRESIS database (i.e. cefepime, cefixime, cefotaxime, cefpodoxime, ceftazidime, ceftibuten and ceftriaxone).

Non-kv-kpc and *K. variicola* isolates showed, respectively, the highest resistance rates against trimethoprim-sulfamethoxazole (9.5% and 3.9%) and against β-lactams (maximum rate for amoxicillin-clavulanic acid: 9.1 and 4.7%), followed by ciprofloxacin (6.9 and 2.1%). Resistance rates against gentamicin, however, only showed a small difference with 2.8% for non-kv-kpc and 1.7% for *K. variicola* isolates, and carbapenem resistance rates were generally low (respectively 0.4 and 0.1%), resulting in broader confidence intervals.

In addition to different resistance rates, *K. variicola* isolates were significantly more often reported from blood and primarily sterile specimens than non-kv-kpc isolates, namely 8.7% *K. variicola* isolates (n = 9,899) and 6.1% of non-kv-kpc isolates (n = 60,215) (Fisher’s exact test: odds ratio = 0.68, 95% confidence interval: 0.63–0.73; p < 0.001).

## Discussion

Our data show that *K. variicola* isolates of the ANRESIS database were significantly more susceptible to six antibiotic classes tested than non-kv-kpc isolates. This is consistent with Cuénod et al., a collaborative international study of several institutions published in 2021 [17]. That study used new generation sequencing data and their own MALDI-TOF spectra to identify eight species of *Klebsiella* within the *K. pneumoniae* and *K. oxytoca* complex and genomic data on the abundance of antimicrobial resistance genes. The authors suggested that higher susceptibility rates in *K. variicola* may be linked to lower abundance of plasmids. 

Differential susceptibility rates may affect surveillance data, as laboratories not differentiating *K. variicola* (in our study accounting for 14.1% of all *K. pneumoniae* complex isolates) tend to underestimate resistance rates of non-kv-kpc isolates. Similar concerns have been raised by a recent review focusing on genomic data of the *K. oxytoca* complex. The authors describe several resistance and virulence genes with the potential to create multidrug-resistant pathogens [18]. Based on data from SENTRY, a global antimicrobial surveillance programme collecting in vitro antimicrobial susceptibility data, the authors observed increasing non-susceptibility rates to carbapenems and cephalosporins over 7 years and highly variable non-susceptibility rates across regions, with higher rates in Asia and Europe than in North America [18]. However, confounding biases as described in our study could not be fully considered due to lack of data. In future large-scale studies comparing different regions and countries, these issues should be addressed to improve our understanding of epidemiological contexts. Similar methodological issues arise in a study by Holt et al. in 2015 [5], showing that the abundance of resistance genes of *K. pneumoniae* isolates from six countries (Australia, Indonesia, Laos, Singapore, the United States and Vietnam) varied between countries. Even within Europe, resistance rates vary considerably, increasing from north to south and from west to east, with Switzerland among the countries with lowest resistance rates [19]. These differences are consistent over time, and even though the confounding bias described in our study may be negligible in some countries, data homogenisation is still needed to increase our understanding of the epidemiological trends in these different species.

Higher resistance rates come with higher abundance of transferable resistance genes [5,17], potentially increasing opportunities for gene transfers between species [20], which may decrease differences in resistance rates between species. This may be especially true in environments where closely related species occur in close proximity [21]. Thus, the impact of an estimation bias of epidemiology and resistance rates may be less severe in countries with higher resistance rates than in countries with lower resistance rates such as Switzerland. Future studies including data from different countries will have to clarify potential differential bias effects between countries.

Studies estimating global health burdens based on statistical modelling or machine learning rely on empirical data of mortality, resistance and virulence genes. These data are obtained from literature reviews, hospitals and surveillance initiatives. Up-to-date knowledge of taxonomy, virulence and antimicrobial resistance is thus crucial to avoid misleading conclusions about the clinical significance or resistance rates of some pathogens [22,23]. Lower susceptibility rates of *K. variicola*, for example, may lead to an underestimation of resistance rates of other members of the *K. pneumoniae* complex and thus to less appropriate treatments of infections. This may affect possible positive effects of appropriately tailored antimicrobial treatments [24].

Our data also indicate that *K. variicola* may be more invasive than non-kv-kpc. However, this association has to be confirmed by clinical and functional studies addressing underlying causes. A recent study by Cuénod et al. comparing different clinical outcomes of patients infected with different *Klebsiella* species did not confirm our data [17], whereas a review of several earlier studies reported associations with blood stream infections in animals and humans [9].

A growing body of data, including our study, suggests that discrimination of species within *K. pneumoniae* complex will increase our knowledge of species-specific susceptibilities and clinical significance, providing valuable information to clinicians and epidemiologists [5,6,9,17]. Correct differentiation of *K. variicola* from the *K. pneumoniae* complex is possible not only by molecular methods such as sequencing of *rpoB*, *fusA*, *gapA*, *gyrA*, *leuS* genes [12,25], but also by updated MALDI-TOF databases used in medical diagnostic laboratories. Since 2017, MALDI-TOF profiles of *K. variicola* have been included in commercial databases allowing identification of *K. variicola*. However, differentiation of *K. variicola* from the *K. pneumoniae* complex by MALDI-TOF is not yet accepted by all microbiological laboratories because the close relatedness of *Klebsiella* spp. prevents verification by 16S rDNA sequencing, a species identification technique used as gold standard by many laboratories. Rodrigues et al. and Cuénod et al. both independently developed MALDI-TOF databases which allow differentiation of all currently known species of the *K. pneumoniae* complex, but discriminatory precision of these two MALDI-TOF databases have not yet been validated using large datasets [17,25]. Continued optimisation of identification methods will be necessary to improve discriminatory power between species of the *K. pneumoniae* complex.

## Conclusion

Higher susceptibility and invasiveness rates of *K. variicola* isolates in our ANRESIS antimicrobial resistance database suggest that refined differentiation of *K. pneumoniae* complex can improve our understanding of its ecological niches, epidemiology, antibiotic susceptibility and clinical significance, thus providing more accurate information to clinicians and epidemiologists. In addition, refined species information has the potential to improve antibiotic prescribing by tailoring antibiotic treatments more appropriately. 

## Supplementary Data

100000 Supplement
