# Supplementary material for: Antibiotic resistance surveillance of Klebsiella pneumoniae complex is affected by refined MALDI-TOF identification, Swiss data, 2017 to 2022
Source: Euro Surveill. 2022 Nov 10;27(45):2200104. doi: 10.2807/1560-7917.ES.2022.27.45.2200104 (PMC9650708; doi:10.2807/1560-7917.ES.2022.27.45.2200104)
Supplement: Supplement [file 22-00104_VOELLMY_Supplement.pdf]

Supplementary material for Eurosurveillance surveillance article "Refined species differentiation of K. pneumoniae complex by modern laboratory methods affects continuous surveillance of antibiotic resistance"

Disclaimer:

This supplementary material is hosted by Eurosurveillance as supporting information alongside the article [Refined species differentiation of *K. pneumoniae* complex by modern laboratory methods affects continuous surveillance of antibiotic resistance], on behalf of the authors, who remain responsible for the accuracy and appropriateness of the content. The same standards for ethics, copyright, attributions and permissions as for the article apply. Supplements are not edited by Eurosurveillance and the journal is not responsible for the maintenance of any links or email addresses provided therein."

Supplementary Table S1: Comparison of non-susceptibility rates of K. variicola and non-kv-kpc strains against six antibiotics using Fisher's exact tests from a total number of 70114 isolates collected by ANRESIS, the Swiss Centre for Antibiotic Resistance, Switzerland, from 1st of January 2017 to 13th of June 2022. P-values are adjusted using sequential Bonferroni corrections. Non-kv-kpc refers to non-*Klebsiella variicola-K. pneumoniae* complex, i.e. to all species of the *K. pneumoniae* complex except *K. variicola*. Carbapenem includes imipenem and meropenem, cephalosporin 3rd/4th generation includes all 3rd and 4th generation cephalosporins available in the ANRESIS database (i.e. Cefepime, Cefixime, Cefotaxime, Cefpodoxime, Ceftazidime, Ceftibuten, Ceftriaxone). 95% CI: 95% confidence interval. Non-susceptible isolates consist of all isolates categorised resistant (R) or intermediate/susceptible, increased exposure" (I) by reporting laboratories.

Antibiotic	non-kv-kpc		*K. variicola*		Odds ratio	95% CI	p-value
	Non-susceptible (%)	n (total)	Non-susceptible (%)	n (total)			
Amoxicillin-clavulanic acid	10.4	60114	5.3	9889	2.1	1.9 - 2.3	< 0.001
Carbapenem	0.5	45460	0.1	7832	4.0	2.1 - 8.5	< 0.001
Cephalosporin 3rd/4th Gen.	6.9	54530	3.1	8883	2.4	2.1 - 2.7	< 0.001
Ciprofloxacin	8.4	59743	3.6	9856	2.4	2.2 - 2.7	< 0.001
Gentamicin	2.8	37806	1.7	6447	1.7	1.4 - 2.0	< 0.001
Trimethoprim-sulfamethoxazole	9.8	58489	4.0	9542	2.6	2.4 - 2.9	< 0.001
